# Central Pontine Myelinolysis Secondary to Hyperglycemia in a Young Patient

**DOI:** 10.7759/cureus.18495

**Published:** 2021-10-05

**Authors:** Wasey Ali Yadullahi Mir, Dhan B Shrestha, Barun B Aryal, Vijay K Reddy, Mir Arshad Ali Yadullahi

**Affiliations:** 1 Department of Internal Medicine, Mount Sinai Hospital, Chicago, USA; 2 Department of Medicine, Mount Sinai Hospital, Chicago, USA; 3 Department of Emergency Medicine, BP Smriti Hospital, Kathmandu, NPL; 4 Department of Neurology, Advocate Christ Hospital, Oak Lawn, USA

**Keywords:** central pontine myelinolysis, osmotic demyelination syndrome, blood glucose, diabetes mellitus, hyperglycemia

## Abstract

Central pontine myelinolysis (CPM) is a neurological disorder typically caused by rapid correction of severe chronic hyponatremia. Conditions causing a hyperosmolar state can also cause CPM, but it is rarely seen in diabetes.

Here we describe a case of a 34-year-old female with longstanding uncontrolled diabetes mellitus who presented with bilateral upper and lower limb weakness and dysphagia. Examination showed decreased muscle strength, and laboratory investigations showed high HbA1c, high blood glucose, increased serum osmolality, and normal sodium. A diagnosis of CPM was made after MRI showed restricted diffusion in the bilateral pons and CT showed pontine hypodensities. The patient was started on insulin therapy, and she showed clinical improvement with improving blood glucose levels. After five days of hospital stay, she was discharged home with appointments to neurology and endocrinology clinics.

This case shows that CPM is a potential complication of uncontrolled diabetes mellitus in the presence of normal serum sodium. Timely treatment of hyperglycemia can lead to improvement of symptoms, but it is a potentially fatal condition. Thus, a diagnosis of CPM should be considered in diabetic patients who present with neurological symptoms and hyperglycemia.

## Introduction

Osmotic demyelination syndrome (ODS) is a rare neurological disorder classically described in the rapid correction of severe hyponatremia [1]. Malnutrition, chronic alcohol usage, and chronic liver disease are known risk factors of ODS [2,3]. ODS typically involves the destruction of myelin sheath in the pons, termed central pontine myelinolysis (CPM), but extrapontine neurons can also be affected, leading to various neurological symptoms. Patients typically present with rapidly progressive neurological symptoms with mental status changes, dysphagia, and dysarthria, but a wide range of presentations have been reported [2-4]. This condition is usually rapidly progressive and potentially fatal. Diagnosis is confirmed by imaging, and magnetic resonance imaging (MRI) is the preferred imaging modality over computed tomography (CT) because of higher sensitivity [3,4]. CT typically shows hypoattenuation, and T2-weighted MRI shows sharply demarcated hyperintensity of the affected areas [3,4]. Management involves supportive care and treatment of the underlying cause.

ODS is rare in hyperglycemic patients with normal sodium levels, though cases have been reported in type 1 and type 2 diabetes mellitus (DM) [5-8]. Here, we present an atypical case of a 34-year-old female with poorly controlled type 2 DM, who presented with a month-long history of weakness and dysphagia and was diagnosed with CPM on imaging. CPM in the absence of hyponatremia and a long progression makes this case particularly interesting.

## Case presentation

A 34-year-old female with a past medical history of type 2 DM presented to the emergency department with a complaint of severe weakness in bilateral upper and lower extremities for one month. The patient reported that her weakness started seven months back when she was hospitalized for Coronavirus disease 2019 (COVID-19). She never completely recovered from this weakness, and over the last one month, it suddenly worsened, affecting her daily activities. The weakness initially involved her hips, then progressed to involve her legs and hands. She also reported difficulty swallowing both solids and liquids over the last month. She denied any recent fever, muscle pain, rash, urinary or stool incontinence, diplopia, slurred speech, or trauma. She also denied any tingling or numbness of extremities. She was diagnosed with type 2 DM during her first pregnancy 13 years before this presentation when she was started on insulin but later transitioned to oral hypoglycemic medications, including metformin, pioglitazone, and glipizide. Family history was significant for DM and lupus in her sister.

On presentation, her vital signs were within normal limits. She was alert and cooperative and well-oriented to time, place, and person. Physical examination showed significant weakness, with motor strength of 4/5 in bilateral upper arms, 3/5 in bilateral lower arms, 2/5 in bilateral hips, and 3/5 in bilateral legs. Sensations were intact, and knee, ankle, and biceps reflexes were 2+. Weakness was more prominent overall in the proximal muscle groups, and there was no muscle tenderness observed. Cranial nerve examination and mental status examination showed no abnormalities.

Laboratory investigations revealed an HbA1c of 12.3%, random blood glucose of 278 mg/dL, and erythrocyte sedimentation rate of 33 mm/h. Her corrected sodium level was 138, and thyroid-stimulating hormone, liver function parameters, lipid profile, complete blood counts, vitamin B12, folate, and creatine phosphokinase were within normal limits. She tested negative for Hepatitis B and C, antinuclear antibodies, anti-double-stranded deoxyribonucleic acid antibodies, and anti-acetylcholine receptor antibodies. Complements C3 and C4 were also within normal limits. Urinalysis showed cloudy urine, many bacteria, positive leukocyte esterase, white blood cell count of 12, and proteinuria. Chest X-rays were normal and did not show any acute cardio-pulmonary processes.

**Figure 1:**
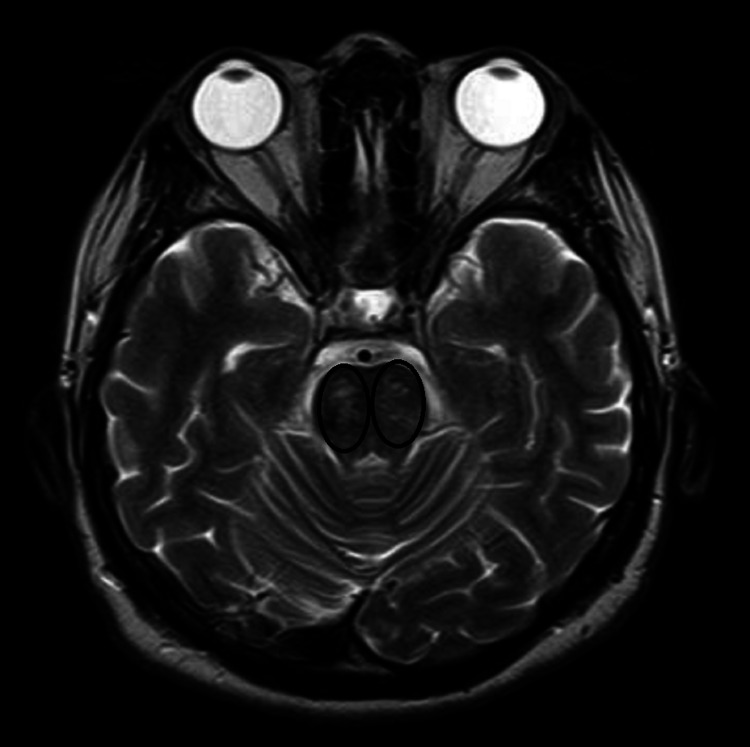
T2/FLAIR axial MR image showing hyperintense lesion over bilateral pons (marked by black circles) FLAIR: Fluid-attenuated inversion recovery

**Figure 2:**
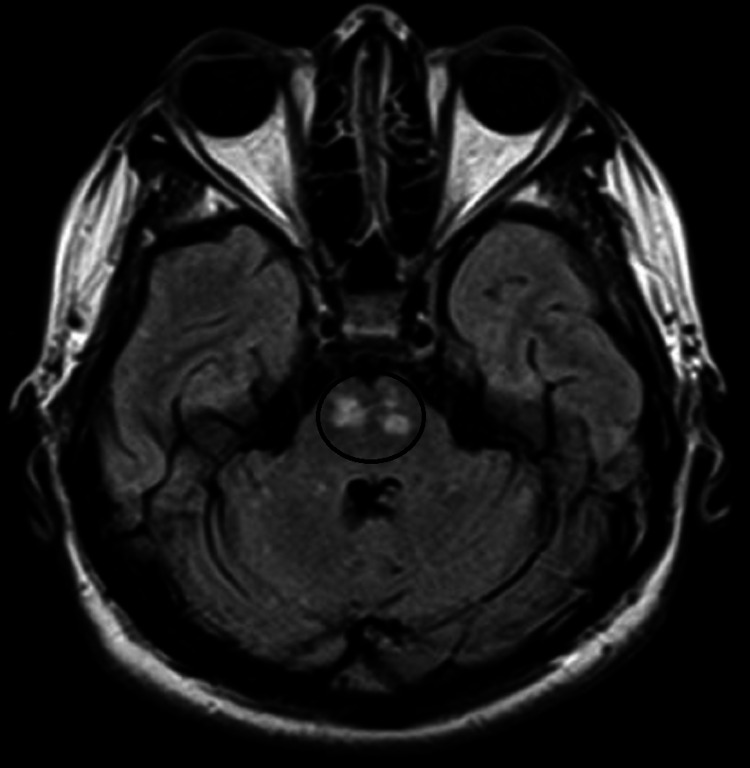
FLAIR axial MR image showing marked hyperintensity over bilateral pons (marked by a black circle) FLAIR: Fluid-attenuated inversion recovery

**Figure 3:**
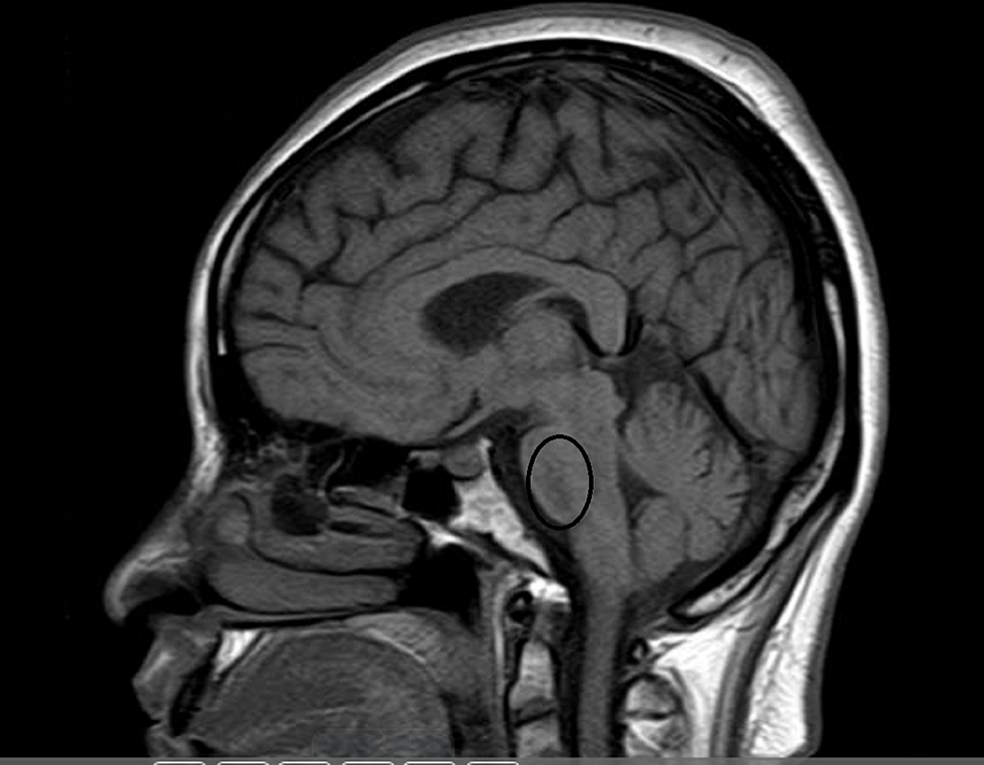
Sagittal section of T1 signal brain MRI image showing mildly hypointense lesion in central pons (marked with a circle)

The patient was started on intravenous methylprednisolone as Guillain-Barre syndrome (GBS) was suspected, and electromyography was planned. On the second day of admission, the patient required a non-diabetic ketoacidosis insulin drip for blood sugar control, likely caused by methylprednisolone. MRI of the brain and cervical spine without contrast showed restricted diffusion in the bilateral pons, making acute ODS more likely than a demyelinating disease (Figures 1-3). The cervical spine was unremarkable on imaging.

The next day, CT was done, which showed pontine hypodensities, corroborating the MRI findings. No acute intracranial results were noted, and there was no large vessel occlusion. The imaging findings were more suggestive of underlying metabolic etiology than demyelination or acute stroke. Methylprednisolone was stopped, and the patient was bridged with 10 units of insulin glargine. She was also started on three units of insulin lispro thrice daily with meals. Despite this, the patient’s blood sugar levels remained in the range of 250 to 300 mg/dL. However, she was showing improvement on her physical examination. Weakness was starting to resolve with 4/5 strength in bilateral upper and lower extremities.

On the fourth day of admission, insulin dosage was increased to 15 units of glargine and five units of lispro thrice daily. She was able to ambulate with the help of physical therapy. The next day, despite a further increase in insulin dosage to 26 units of glargine and 11 units of lispro thrice daily, her blood sugar levels remained above 200 mg/dL. She could get up from bed and walk on her own but needed to rest after a short period of activity. The patient was then discharged home with instructions to follow up with a neurologist on an outpatient basis.

## Discussion

Central pontine myelinolysis was first described in 1959 in patients with quadriparesis and pseudobulbar palsy, who were shown to have demyelination confined to the central area of the pons [2]. It was initially linked to malnutrition and alcoholism, and the association with rapid correction of severe chronic hyponatremia was established nearly two decades later [9]. Shrinkage of oligodendrocytes in response to increasing osmolality is the pathogenesis behind osmotic demyelination [9]. While hyponatremia is the major predisposing factor, conditions like hypokalemia, other electrolyte disturbances, liver cirrhosis, severe burns, and acquired immunodeficiency syndrome have also been implicated in this condition by recent studies [2,3,10]. ODS is a term that encompasses both the pontine and extrapontine demyelination caused by hyperosmolality.

We have described a case of central pontine myelinolysis in a young female with type 2 diabetes mellitus for 13 years. She was under oral hypoglycemic drugs, but her HbA1c of 12.3 and persistently elevated blood glucose levels proved that her blood glucose was not well-controlled. The patient first noticed weakness of limbs seven months before presentation, when she was diagnosed with COVID-19, which required hospitalization. She had residual weakness since then, and it increased in severity during the previous month. Sodium and other electrolyte levels were normal at presentation other than blood glucose at 278 mg/dL. The differential diagnosis initially included demyelinating disease, post-viral myopathy, myasthenia gravis, and GBS. She was immediately started on intravenous steroids. The diagnosis of CPM was only reached after brain imaging showed characteristic findings. Treatment with insulin improved her symptoms, and she was discharged on the fifth day of admission.

Uncontrolled blood glucose can lead to many significant complications in diabetic patients, and CPM is one such potentially fatal complication. As the serum sodium level was normal and she had marked and likely longstanding hyperglycemia, hyperglycemia was the most probable cause of pontine myelinolysis in this patient. Increased blood glucose leads to increased serum osmolality, and pontine myelin sheath destruction is a potential sequela of a hyperosmolar state. Typically, CPM is described as rapidly progressive, and it causes mental status changes and dysarthria [3,4]. However, the patient had an intact mental status in this case, and dysphagia was the only significant bulbar symptom. Moreover, slowly progressive weakness over a month is highly unusual.

As sodium is the major contributor to serum osmolality, it is expected to be the most common culprit for hyperosmolality. But alternative causes of hyperosmolality must be sought in cases of osmotic demyelination with normal sodium levels. Hyperosmolality can often be seen with hyperglycemia. However, ODS has rarely been described in the context of diabetes mellitus. There have been cases of hyperglycemic patients developing ODS, both associated with rebound hypernatremia [5,11] and with normal sodium levels [6,8,12-15]. In 2012, Hegazi and Mashankar reported the case of a diabetic female with CPM in association with hypernatremia caused by a hyperosmolar hyperglycemic state (HHS) who was treated conservatively [5]. Another case of CPM with hypernatremia complicating the management of HHS was reported by O’Malley et al. in 2008 [11]. Hyperglycemia is associated with decreased serum sodium levels and thus can be a contributing factor in the typical presentation of osmotic demyelination with correction of hyponatremia. Rarely, however, it can also be a standalone cause of ODS. In 2016, Donnelly et al. reported the case of CPM in a young male with type 1 diabetes with normal sodium levels [6]. The patient presented with slurred speech, dysphagia, and nasal regurgitation, different from our case presentation. His symptoms improved over weeks but did not resolve completely, and his prognosis was further worsened by end-organ damage due to diabetes.

In contrast to this, Talluri et al., in 2017, reported a case of CPM in a 45-year-old male with type 2 diabetes, who presented with gait imbalance, slurred speech, and inappropriate laughter. He was also hyperglycemic and normonatremic, but in eight weeks, his symptoms showed near-complete resolution [8]. Consistent with our case, studies have shown that pontine involvement is common in patients with ODS. However, cases with extrapontine demyelination have also been reported by Guerrero et al. and Hirosawa and Shimizu [13,15]. However, these two cases had different outcomes. Guerrero et al. reported no improvements in symptoms and Hirosawa and Shimizu reported complete resolution of symptoms, albeit with persistent abnormalities on imaging. This highlights the great variation in the prognosis of CPM in the context of hyperglycemia. Typically, patients present with a few days or a week of acute progressive quadriparesis, dysphagia, and dysarthria. Mental status changes such as confusion and delirium are often seen. The major difference between the cases described in the literature and the current case is the slower progression of symptoms in the latter.

Recently, with the emergence of COVID-19, there have been several case reports and clinical encounters where ODS/CPM was reported [16,17]. However, all those were reported in the background of rapid correction of hyponatremia, which was not in our case though the patient was diagnosed for COVID-19 earlier.

There is no specific treatment for ODS, and the management is mostly symptomatic. Removal of the underlying cause of hyperosmolality is important for treatment. In our case, even though hyperglycemia was not completely corrected early on, the patient’s symptoms improved with a decrease in serum glucose and resolved within a few weeks. This highlights the importance of correction of blood glucose in the management of patients with ODS due to hyperglycemia.

## Conclusions

Central pontine myelinolysis can occur due to hyperglycemia even with normal serum sodium. Therefore, a diagnosis of CPM should be considered in diabetic patients with poor glycemic control who present with neurological symptoms like quadriparesis, even in the absence of derangements of serum sodium or other predisposing factors. Early diagnosis with imaging and treatment of the underlying cause, i.e., hyperglycemia, can lead to rapid symptomatic improvement.
